# 1267. Efficacy and Safety of Switching to DTG/3TC in Virologically Suppressed PLWH by Age, Including Those Aged ≥65 Years: Pooled Results from the TANGO and SALSA Studies

**DOI:** 10.1093/ofid/ofac492.1098

**Published:** 2022-12-15

**Authors:** Manyu Prakash, Richard Grove, Brian R Wynne, Choy Man, Jean A van Wyk, Clifford B Jones, Chinyere Okoli, Emilio Letang, Mounir Ait-Khaled, Andrew Clark

**Affiliations:** ViiV Healthcare, Brentford, UK, Brentford, England, United Kingdom; GlaxoSmithKline, Brentford, UK Brentford, England, United Kingdom; ViiV Healthcare, Research Triangle Park, NC, USA, Chagrin Falls, Ohio; ViiV Healthcare, Research Triangle Park, NC, USA, Chagrin Falls, Ohio; ViiV Healthcare, Brentford, UK, Brentford, England, United Kingdom; ViiV Healthcare, Brentford, UK, Brentford, England, United Kingdom; ViiV Healthcare, Brentford, UK, Brentford, England, United Kingdom; ViiV Healthcare, Madrid, Spain, Madrid, Madrid, Spain; ViiV Healthcare, Brentford, UK, Brentford, England, United Kingdom; ViiV Healthcare, Brentford, UK, Brentford, England, United Kingdom

## Abstract

**Background:**

The proportion of older adults living with HIV is growing, increasing the focus of managing age-related comorbidities and polypharmacy while maintaining virologic suppression. DTG/3TC is an international guidelines–recommended 2-drug regimen demonstrating high and durable efficacy, high barrier to resistance, and good safety and tolerability. We present pooled TANGO and SALSA efficacy and safety results analyzed by age (< 50, ≥ 50 to < 65, and ≥ 65 years).

**Methods:**

Week 48 data from the open-label phase 3 TANGO and SALSA trials evaluating switch to once-daily DTG/3TC fixed-dose combination or continuing current antiretroviral regimen (CAR; TANGO: TAF-based; SALSA: NNRTI-/bPI-/INSTI-based) were pooled. Proportions of participants with HIV-1 RNA ≥ 50 and < 50 c/mL (Snapshot, ITT-E) and safety were analyzed by age.

**Results:**

Of 1234 participants (DTG/3TC, n=615; CAR, n=619), 71% were aged < 50 years, 26% aged 50 to < 65 years, and 3% aged ≥ 65 years. At Week 48, in participants aged 50 to < 65 years, 1 (< 1%) and 3 (2%) participants in the DTG/3TC and CAR groups, respectively, had HIV-1 RNA ≥ 50 c/mL, comparable to results in the overall population (Table 1). No participants aged ≥65 years had HIV-1 RNA ≥ 50 c/mL. Proportions with HIV-1 RNA < 50 c/mL were high regardless of age group. Adjusted mean change from baseline in CD4+ cell count and CD4+/CD8+ ratio was also consistent with overall results regardless of age (< 50 vs ≥ 50 years). No participants in the DTG/3TC group had confirmed virologic withdrawal (CVW); 1 participant in the CAR group had CVW (aged < 50 years; no resistance detected). Proportions of participants with baseline non-ART medication use and comorbidities increased with age in both treatment groups (Table 2). Across all age groups, proportions of any AEs were similar between the DTG/3TC and CAR groups, with few AEs leading to withdrawal and higher proportions of drug-related AEs with DTG/3TC vs CAR; similar results were observed in the overall population.

**Figure:**
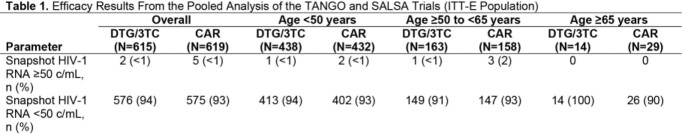

**Figure:**
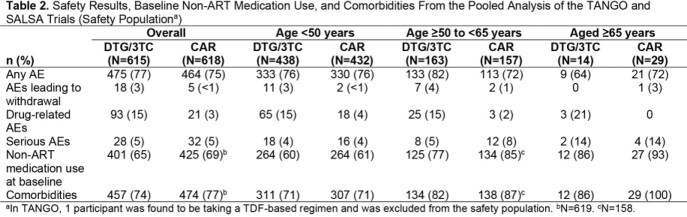

**Conclusion:**

Among PLWH, switching to DTG/3TC maintained high rates of virologic suppression and demonstrated a favorable safety profile across all age groups, including those aged ≥65 years. As non-ART medication use and comorbidities increased with age, a well-tolerated 2-drug regimen may help older PLWH avoid drug interactions.

**Disclosures:**

**Manyu Prakash, PhD**, GlaxoSmithKline: Stocks/Bonds|ViiV Healthcare: Employee **Richard Grove, MSc**, GlaxoSmithKline: Employee|GlaxoSmithKline: Stocks/Bonds **Brian R. Wynne, M.D.**, ViiV Healthcare: I am an employee|ViiV Healthcare: Stocks/Bonds **Choy Man, BSc**, GlaxoSmithKline: Stocks/Bonds|ViiV Healthcare: Employee **Jean A. van Wyk, MBChB, MFPM**, GlaxoSmithKline: Stocks/Bonds|ViiV Healthcare: Employee **Clifford B. Jones, BSc MSc MB ChB**, GSK: Stocks/Bonds|viiv healthcare: Employee **Chinyere Okoli, MSc, DIP**, ViiV Healthcare: I am an employee of ViiV healthcare|ViiV Healthcare: Stocks/Bonds **Emilio Letang, MD, MPH, PhD**, GlaxoSmithKline: Stocks/Bonds|ViiV Healthcare: Employee **Mounir Ait-Khaled, PhD**, GlaxoSmithKline: Stocks/Bonds|ViiV Healthcare: Employee **Andrew Clark, MD**, ViiV Healthcare: Employee|ViiV Healthcare: Stocks/Bonds.

